# Balancing conflicting mitigation and adaptation behaviours of urban residents under climate change and the urban heat island effect

**DOI:** 10.1016/j.scs.2020.102585

**Published:** 2021-02

**Authors:** Kayoko Kondo, Leslie Mabon, Yifan Bi, Yulin Chen, Yuriko Hayabuchi

**Affiliations:** a Department of Environmental Design, Faculty of Design, Kyushu University, Japan; b School of Applied Social Studies, Robert Gordon University, UK; c Faculty of Design, Graduate School of Design, Kyushu University, Japan; d Global Innovation Center, Kyushu University, Japan

**Keywords:** Air conditioning, Climate change communication, Cooling behaviours, Urban climate change, Urban heat island

## Abstract

Under a warming climate and urban heat island effects, cooling behaviours are increasingly important for city dwellers. Cooling actions, especially air conditioning, receive increasing scrutiny in social science, as does engagement and communication on behaviours spanning adaptation and mitigation. In response, this paper evaluates the relation between residents’ adaptation and mitigation behaviours around cooling in Fukuoka, Japan, and draws lessons for communication on encouraging adaptation and mitigation actions. A survey distributed to residents in six areas of Fukuoka, Japan, assessed perceptions of global warming and urban heat island effects, frequency of mitigation and adaptation behaviours, use of air conditioning, electricity bills and evaluation of green spaces. We observe a difference between respondents using air conditioning with an energy-saving (i.e. mitigation) focus, versus those using air conditioning with an adaptation (i.e. cooling) focus. We also note residents emphasising mitigation behaviours may use shade in parks or cooling centres as alternative cooling strategies, but that awareness of effective air conditioning use may be lacking. Our findings build on existing literature by reinforcing – in a subtropical context – the need to reconsider practices around air conditioner use; and illustrate the value of a breadth of messages to promote joint mitigation and adaptation actions.

## Highlights

• Cooling behaviours an area of increasing interest under rising heat in cities.
• Survey of public in Fukuoka, Japan, assesses cooling behaviour and climate awareness.
• Some people use air conditioning with mitigation focus, others emphasise adaptation.
• Those emphasising mitigation have lower bills and more likely to engage in other behaviours.
• Messaging on air conditioning use and promoting urban greening may enable co-benefits.

## 1 Introduction

In cities, risks to urban dwellers from increased heating under climate change are exacerbated by the urban heat island effect, whereby processes of urbanisation compound existing warming trends. Yet whilst there is a large body of research on urban heat islands and their effects on people for temperate climates, scholarship on urban thermal environments for lower latitudes is still emerging (Giridharan & Emmanuel, 2018) and has indeed been called for in outputs affiliated with the Intergovernmental Panel on Climate Change (Prieur-Richard et al., 2018). The purpose of this paper is to contribute to this emergent understanding through evaluation of citizens’ cooling practices in Fukuoka, Japan. We look in particular at citizens’ use of air conditioning as a climate change adaptation measure, and how this use of air conditioning may relate to citizens’ climate change mitigation practices. Through doing so, we aim to contribute to social science theorisations of the links between cooling, practice, and socio-cultural contexts, and to thought on climate change communication at the mitigation-adaptation interface. These issues are evaluated through a survey conducted with residents in Fukuoka, which assessed their adaptation and mitigation practices and their environmental concerns more widely In terms of existing international literature, this work contributes to understandings of individuals’ cooling practices as being located within a wider social and cultural context.

Under a warming urban environment, air conditioning has both benefits and drawbacks. On one hand, air conditioning is an important cooling and adaptation strategy for city dwellers, and can especially reduce risks from excess heat for vulnerable groups such as the elderly or those with pre-existing medical conditions (Bao, Li, & Yu, 2015; Leal Filho, Echevarria Icaza, Neht, Klavins, & Morgan, 2018). Yet the use of air conditioners also increases energy consumption – hence potentially contributing to climate change - and further raises the outdoor temperature due to exhaust heat from the outdoor unit of the air conditioner (Ihara, Kikegawa, Asahi, Genchi, & Kondo, 2008; Mushore, Odindi, Dube, & Mutanga, 2017; Solecki et al., 2005).

Moreover, whilst research has shown that low-income households may be more concerned about health risks from heat and climate change, low-income households in the US were less likely to have air conditioning at home (Madrigano et al., 2018). Lack of transportation or lack of awareness can prevent the most vulnerable people from accessing and using public cooling centres (Nayak et al., 2017). Concern over climate change – and a sense of responsibility for changing behaviours in response – has been found to be high for social housing tenants in the UK (Hayles & Dean, 2015). In recent years, the Japanese government has started to discuss establishing a grant for buying air conditioning and covering electricrity costs, and has also considered using public facilities such as public halls as cool sharing spots (Ministry of Environment, 2020).

Partly in response to this increasing concern with air conditioning in cities, there has been recent engagement with the social and cultural contexts governing people’s relationships with cooling technologies and cooling behaviours. Shove, Walker, and Brown (2014) ask how the necessity of air conditioning becomes embedded within different practices, and how such practices may change over time. Walker, Shove, and Brown (2014) advocate for considering the necessity of air conditioning in relation to not only practices, but also discourses (such as health and wellbeing) and institutional arrangements. Emphasising the social nature of cooling behaviours, Winter (2016): 430) calls for research into climate change and mechanical cooling to reimagine people as “active participants in the attainment of thermal comfort.” Nonetheless, Sampson et al. (2013) find that financial, cultural or knowledge barriers can hinder those who would benefit most from air conditioning in using it properly, and remind that air conditioning may be one of only a number of cooling behaviours people adopt.

There is hence a body of extant research into how air conditioning is embedded within individuals’ behaviours, actions, and lifestyles. However, much of this work comes from temperate climate contexts such as the United Kingdom and parts of the USA, and has an emphasis on trying to understand people’s practices as a means of stopping the ‘spread’ of air conditioning and hence mitigating climate change. By contrast, in tropical and subtropical city contexts, air conditioning is already widespread and may – if used sensitively – form part of a suite of adaptation measures for climate changes that cities are already locked into (e.g. Leal Filho et al., 2018). In this study, we therefore aim to understand (a) how citizens understand cooling behaviours such as air conditioning in relation to climate change mitigation; and (b) how this relates to citizens’ awareness of air conditioning and wider cooling behaviours as an adaptation strategy.

Our work is guided by the emergent body of research into the interface between mitigation and adaptation actions, specifically research into the kinds of communication and messaging strategies that may help citizens engage in not only mitigation behaviours but also appropriate adaptation actions. At the city-wide level, there has been interest in linking mitigation and adaptation approaches (Thornbush, Golubchikov, & Bouzarovski, 2013), especially ‘co-benefits’ when understood as mitigation and adaptation win-wins alongside wider environment and development benefits (Puppim De Oliveira, 2013). Bain et al. (2016) suggest that communication of such co-benefits can motivate action at personal level for both those concerned by climate change and those who are not.

Moreover, Demski, Capstick, Pidgeon, Sposato, and Spence, 2017: 161) hold that “climate change communication is often framed solely as a matter of fostering mitigation action. But, in the face of rising climate risks, support for climate related policies and the nature of public adaptation responses will become increasingly important for social sciences research.” Work to date indicates that mitigation actions may be more engaging to people with high awareness of or concern for environmental issues, whereas adaptation actions or rationales grounded in economic benefits may be more engaging to those with less concern for climate change (Howell, Capstick, & Whitmarsh, 2016; Nauges & Wheeler, 2017). There is also need in communication to understand and account for maladaptive behaviours, such as urban greening for cooling purposes triggering allergies (Escobedo, Kroeger, & Wagner, 2011) or inappropriate use of air conditioning (Sampson et al., 2013).

Our study therefore takes this question of how citizens understand cooling behaviours in relation to mitigation and adaptation, and uses it to contribute not only to conceptual understandings of cooling practices in a context where air conditioning is also prevalent, but also to make an empirical contribution to understandings of messaging, communication and engagement at the mitigation-adaptation interface. To do so, we evaluate citizen behaviours in the subtropical city context of Fukuoka, Japan.

## 2 Research details

### 2.1 Method

**Fig. 1:**
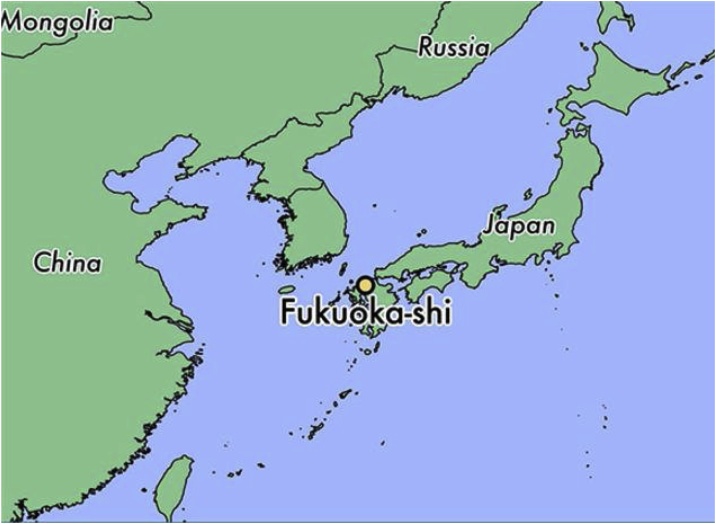
Location of Fukuoka City in Japan.

This study focused on Fukuoka City, which is located in the Kyushu area in Japan (Fig. 1). Fukuoka has a population of 1.5 million and a humid subtropical climate. Fukuoka is experiencing a faster rate of warming than the rest of Japan (Mabon, Kondo, Kanekiyo, Hayabuchi, & Yamaguchi, 2019), and the city government lists the urban heat island as one of the key adaptation challenges for the city within their climate change plan (Fukuoka City, 2016). The city has recorded an almost year-on-year rise in cases of heatstroke in recent years, with 821 emergency callouts for heatstroke in 2018 (Fukuoka City, 2019a). The establishment of ‘cool share’ facilities (public spaces where citizens can access air conditioning on hot days) and initiatives to encourage citizens to facilitate cooling via growing of green curtains (Fukuoka City, 2016) and scattering of water on pavements (Fukuoka Prefecture, 2019) also illustrate the seriousness with which authorities in Fukuoka are taking the issue of urban heat.

A questionnaire survey on global warming and the urban heat island effect was carried out with Fukuoka residents in January 2018. The survey included respondents’ perception of the problem, evaluation of measures, and mitigation and adaptation behaviours. As respondents use public transport, parks/green spaces, and materials for greening their home, their behaviour is influenced by geographical conditions and housing situation. Six areas were surveyed to reflect three key areas of enquiry: use of public transportation, proximity to greenspace, and type of housing. As the use of public transport as a mitigation option and the use of greenspace as a cooling strategy were areas of enquiry in the survey, sampled areas were selected to give one area near to and one area far from a subway station, and one area near to and one area far from greenspace. Moreover, as behaviours such as greening may differ between apartments and detached houses, one area with many apartments (in the city centre) was surveyed, and one area with many detached houses (in the suburbs) was surveyed (see Table 1). A total of 1,000 questionnaires were distributed to households and collected by post. The questionnaires were analysed through comparison of means, factor analysis, correlation analysis and multiple regression analysis.

**Table 1:** Distribution area of survey.

	Distribution area	
	Apartment building	Detached house
Inner city	Akasaka	Ropponmatsu
	Nishijin	Befu
Suburbs	Rouji	Rouji
	Shiohama	Shiohama

### 2.2 Survey items and respondents’ attributes

The questionnaire covered five areas: 1. Perception of global warming and the urban heat island effect; 2. Current mitigation and adaptation behaviours being carried out; 3. Evaluation of green spaces, 4. Length of time per a day and number of months over which air conditioner is used during summer, and 5. Electricity bill for August as the hottest month of the year. It is of course true that mitigation and adaptation are not entirely separate, and that some actions may contribute to both mitigation and adaptation. We thus adopted the IPCC definitions of mitigation (“An anthropogenic intervention to reduce the sources or enhance the sinks of greenhouse gases” (Klein et al., 2007: 750)) and adaptation (“Adjustment in natural or human systems in response to actual or expected climatic stimuli or their effects, which moderates harm or exploits beneficial opportunities” (Klein et al., 2007: 750)) to distinguish between the two categories. For the purposes of this study, we therefore classified *mitigation behaviours* as those where the primary intention is to reduce the extent of climate change or urban heat island effects in the near-term and long-term future by taking actions that reduce the amount of electricity consumed and hence the emissions produced. Conversely, we classified *adaptation behaviours* as those where the primary intention is to reduce the impacts of rising temperatures that are already happening.

Table 2 lists the mitigation and adaptation behaviours under the urban heat island effect/global warming. Respondents were asked which behaviours they carry out over a 5-point scale, with 1 being never and 5 being all the time. Questions on mitigation behaviours consist of 15 kinds that are effective in reducing greenhouse gas emissions and energy consumption directly or indirectly. Questions on adaptation behaviours consist of 11 kinds that reduce the sensible temperature and heat illness directly or indirectly.

**Table 2:** List of mitigation behaviors and adaptation behaviors.

Mitigation behaviors	Adaptation behaviors
1. Using public transportation	1. Having fluid frequently
2. Setting temperature of air conditioner to 28℃ in summer	2. Paying attention to salt absorption
3. Setting temperature of air conditioner to 20℃ in winter	3. Reducing room temperature appropriately using fan or air conditioning
4. Unplugging household appliances that are not used	4. Adjusting air conditioner to right temperature to ensure comfortable sleeping environment
5. Regarding energy efficiency as criterion when making purchasing decisions	5. Paying attention to temperature and humidity
6. Closing curtains and blinds when using air conditioner	6. Dressing appropriately for weather
7. Avoiding direct sunlight and ventilation	7. Avoiding going out on hot days
8. Using "green curtains"	8. Bringing drink when going out
9. Increasing vegetation	9. Using hat or parasol under sunshine
10. Sprinkling water	10. Taking frequent rest in open air on hot days
11. Using reed screen	11. Staying under shade of trees
12. Using window glass with good thermal insulation	
13. Using LED	
14. Trying "cool share"	
15. Staying in living room when using air conditioner	

**Fig. 2:**
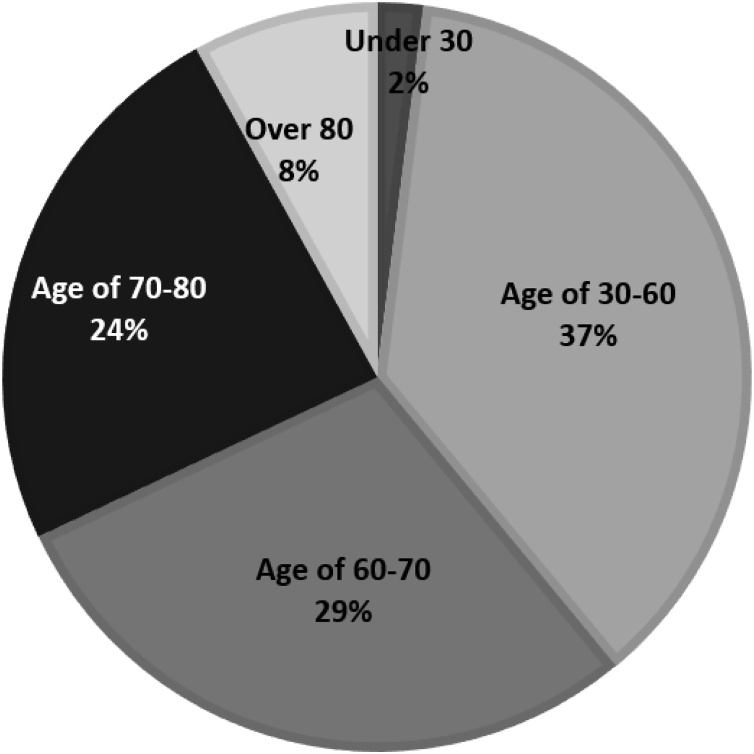
Age composition of respondents.

The number of returned valid questionnaires was 246 (valid collection rate: 24.6 per cent). In terms of gender, respondents were 53.4 per cent male and 46.6 per cent female. As for age, respondents under 30 accounted for 2 per cent, respondents in their 30 s to 50 s represented 37 per cent, respondents in their 60 s accounted for 29 per cent, those in their 70 s made up 24 per cent of the sample and those over 80 accounted for 8 percent (Fig. 2). As for housing situation, 41.3 per cent of the respondents lived in collective housing (i.e. apartment blocks) and 58.7 per cent in detached houses.

According to the 2019 Fukuoka City Statistics Handbook, for Fukuoka City as a whole, the population is 47.2 per cent male and 52.8 per cent female; 31% under 30, 41% in their 30 s to 50 s, 11% in their 60 s, 10% in their 70 s, and 7% in 80 s and up. Whilst available data does not provide a split between apartment blocks and detached houses, 50% of households live in privately rented accommodation, and a further 12% live in public, housing authority or company housing which are likely to be apartment-style (all figures from Fukuoka City, 2019b). Our sample therefore contains more male respondents, an older pool of respondents, and potentially more people living in detached houses than might be expected for a truly ‘representative’ sample of Fukuoka City. However, as the purpose of the study is to make a contribution to scholarly knowledge on drivers of mitigation and adaptation behaviours under increasing temperatures, and not to generalise to Fukuoka City as a whole or interpret our findings in light of characteristics specific to Fukuoka, we believe the sample returned can still yield valuable analytical insights.

## 3 Results

### 3.1 Mitigation and adaptation behaviours

**Fig. 3:**
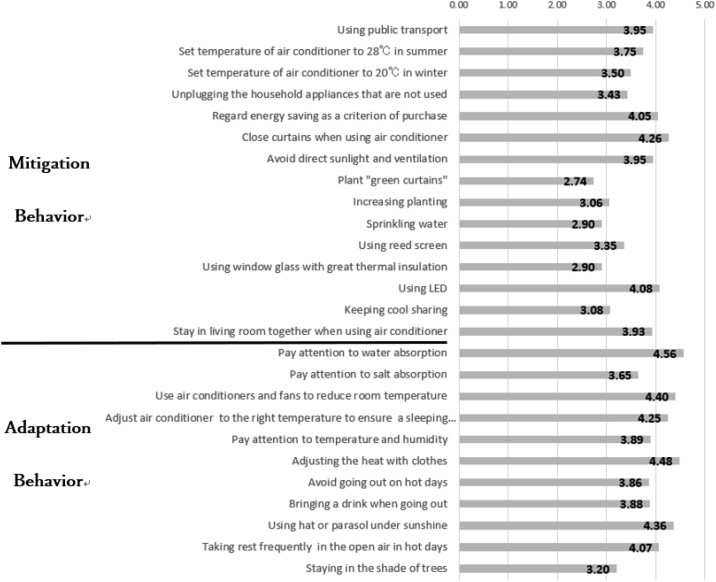
Average frequency of mitigation and adaptation behaviors (0=never; 5=always).

Fig. 3 shows the means of mitigation and adaptation behaviours that respondents undertake. Among the mitigation behaviours, the behaviours that respondents most often carry out are ‘close the curtains when using air conditioning (4.26)’, ‘use LED (4.08)’, and ‘regard energy-saving as a criterion of purchase (4.05)’. ‘Set temperature of air conditioner’at the energy-saving level scored lower than the above three behaviours. ‘Using window glass with great thermal insulation’ (2.90), ‘sprinkling water’ (2.90), and ‘plant “green curtains”’ (2.74) had the lowest scores.

Among adaptation behaviours, the behaviours that respondents most often carry out are, in descending order: ‘pay attention towater absorption (4.56)’, ‘Adjust the heat with clothes (4.48)’, ‘use air conditioners and fans to reduse room temperature (4.40)’, and ‘use hat or parasol under sunshine (4.36)’.

### 3.2 Factor structure of mitigation and adaptation behaviours

As there were many kinds of mitigation and adaptation behaviours, synthesis variables were created by means of factor analysis (Table 3). We performed factor analysis by the main factor method for each mitigation behaviours and adaptation behaviours, and performed varimax rotation.

**Table 3:** Factor analysis of mitigation behavior and adaptation behavior.

		Items	Factor loading
Mitigation Behavior	1. Use of natural elements	Increasing vegetation	0.713
		Using "green curtains"	0.663
		Sprinkling water	0.632
		Using reed screen	0.581
	2. Energy-saving use of air conditioning	Setting temperature of air conditioner to 28℃ in summer	0.703
		Setting temperature of air conditioner to 20℃ in winter	0.627
		Avoiding direct sunlight and ventilation	0.543
		Trying "cool share"	0.499
		Staying in living room when using air conditioner	0.473
	3. Energy-saving through habitual actions	Closing curtains and blinds when using air conditioner	0.677
		Regarding energy efficiency as criterion when making purchasing decisions	0.553
		Unplugging household appliances that are not used	0.511
	4. Purchase of energy-saving goods	Using window glass with good thermal insulation	0.543
		Using LED	0.499
		Having window grille or a shutter in the bedroom	0.379
	5. Public transport	Using public transportation	0.555
Adaptation Behavior	1. Use of air conditioning and cooling approaches	Using air conditioners and fans to reduce room temperature	0.788
		Dressing appropriately for weather	0.486
		Adjusting air conditioner to right temperature to ensure comfortable sleeping environment	0.485
		Paying attention to temperature and humidity	0.466
	2. Fluid/salt intake and rest	Paying attention to salt absorption	0.737
		Having fluid frequently	0.586
		Bringing drink when going out	0.467
		Taking frequent rest in the open air in hot days	0.535
	3.Shade preference	Using hat or parasol under sunshine	0.441
		Staying under shade of trees	0.394
		Avoiding going out on hot days	0.307

The first factor in mitigation behaviours was made up of variables that lower environmental load by using nature, such as increasing vegetation and creating shade; this factor was named ‘use of natural elements’. The second factor was made up of variables related to energy-saving when using air conditioning, such as temperature setting, ‘cool share’, shutting off of sunlight, and having better ventilation; this factor was named ‘energy-saving use of air conditioning’. The third factor was made up of everyday energy-saving behaviours, such as the use of curtains, purchase of energy-saving electrical appliances, and plugging off; this factor was named ‘energy-saving through habitual actions’. The fourth factor was named ‘purchase of energy-saving goods’ because it was made up of the purchase of thermal-insulated windows and LEDs, and the installation of grilles or shutters in the bedroom. The fifth factor was made up of one variable; it was named ‘public transport’.

The first factor in adaptation behaviour was named ‘use of air conditioning and cooling approaches’ as it was made up predominately of air conditioning and cooling-related behaviours: ‘lowering the temperature with an appropriate use of air conditioning’, ‘adjusting to the temperature using appropriate clothing’, and ‘the use of air conditioning while sleeping’ (plus also ‘appropriate clothing’). The second factor was named ‘fluid/salt intake and rest’ and consisted of factors to preserve physical energy: ‘frequent intake of fluid/salt’ and ‘taking frequent rest’. The third factor was named ‘shade preference’ as it was made up of ‘using a hat or parasol’, ‘staying under the shade of trees’ and ‘avoid going out’.

**Fig. 4:**
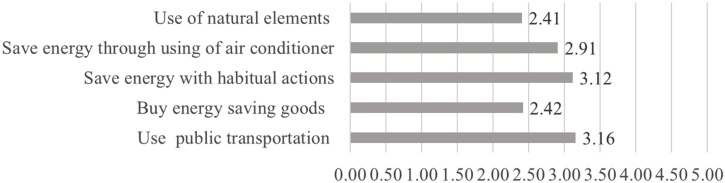
Average frequency of mitigation behavior factors (0=never; 5=always).

**Fig. 5:**
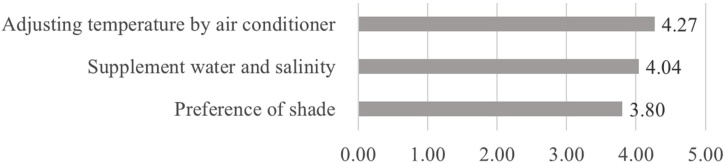
Average frequency of adaptation behavior factors (0=never; 5=always).

Fig. 4, Fig. 5 show comparison of the means by factors in mitigation/adaptation behaviours. The mitigation behaviour with the highest score was ‘public transport (3.16)’, followed by ‘energy-saving by habitual actions (3.12)’ and ‘energy-saving by using air conditioning (2.91)’. The behaviours with low scores were ‘the purchase of energy-saving appliances (2.42)’ and ‘the use of natural elements (2.41)’. The most frequently deployed mitigation behaviors are hence actions that require the least additional work. The adaptation behaviour with the highest score was ‘the use of air conditioning (4.27)’, followed by ‘frequent intake of fluid/salt (4.04)’ and ‘preference for shade (3.80)’. Likewise, the most frequently deployed adaptation behaviour (air conditioning) is the one that is most reliant on technology as opposed to changes in individual habits or routines.

### 3.3 Relationship between each factor of mitigation and adaptation behaviours and the use of air conditioning in summer and electricity bill

Regarding the use of air conditioning, there is a contrast between mitigation and adaptation behaviour. Using air conditioning with a ‘mitigation’ motivation encourages restriction of the use to save energy, whereas using air conditioning with an ‘adaptation’ motivation promotes use to maintain health and comfort against rising temperatures. Consequently, a correlation analysis was conducted among each factor of mitigation/adaptation behaviour, the length and period of use of air conditioning in early summer and mid-summer, and the electricity bill for August (Table 4). For the length of use of air conditioning, respondents were asked about the total length of use per day and whether they used air conditioning during sleep.

**Table 4:** Correlation analysis of mitigation behavior and adaptation behavior factors.

		Use of natural elements	Saving energy through use of air conditioner	Saving energy with habitual actions	Buying energy saving goods	Using public transportation	Adjusting temperature by using air conditioner	Supplementing water and salinity	Preference of shade
Time of using air conditioner	Early June daytime	−0.039	-.286**	−0.039	0.083	0.026	.207**	0.006	−0.094
	Early June sleeping time	0.057	−0.106	−0.032	0.057	0.006	.140*	0.114	−0.040
	Early August daytime	−0.005	-.254**	0.012	0.009	−0.005	.352**	0.039	−0.093
	Early August sleeping time	−0.083	-.151*	−0.018	−0.067	−0.034	.295**	0.112	−0.040
Period of using air conditioner		−0.110	-.294**	−0.114	0.048	0.056	.210**	0.114	−0.104
Electricity bill in August		−0.035	-.186**	-.232**	0.045	0.062	−0.020	−0.015	-.190**

**p < 0.01.

*p < 0.05.

As Table 4 shows, all factors, except for the length of use of air conditioning during sleep in early June (early summer), were significantly correlated to ‘an energy-saving use of air conditioning’ (i.e. using air conditioning with a mitigation motivation). There was negative correlation with the length of use of air conditioning while sleeping in early August (r = -0.151, *p* < 0.05), periods of use of air conditioning (r = -0.294, *p* < 0.01), and electricity bill for August (r = -0.186, *p* < 0.01). There is negative correlation between ‘energy-saving by habitual actions’ (r = -0.232, *p* < 0.01) and ‘shade preference’ (r = -0.190, *p* < 0.01) and electricity bill for August. In short and perhaps unsurprisingly, these results suggest that those practicing energy-saving uses of air conditioning are likely to use air conditioning for less time, and to see lower electricity bills in summer.

Except for the electricity bill for August, ‘use of air conditioning and cooling approaches’ was positively correlated with the length of use per day at the beginning of June (early summer) (r = 0.207, *p* < 0.01), sleeping at the beginning of June (early summer) (r = 0.140, *p* < 0.05), per day at the beginning of August (mid-summer) (r = 0.352, *p* < 0.01), and while sleeping at the beginning of August (mid-summer) (r = 0.295, *p* < 0.01). In other words, those using air conditioning with ‘adaptation’ motivations were likely to use air conditioning for more time each day, and over a longer number of months during summer.

### 3.4 Comparing the amount of electricity used

**Fig. 6:**
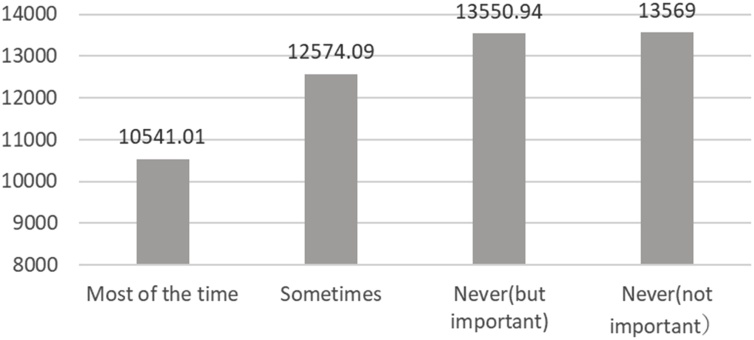
Relation between how often respondents carry out action of “Setting the cooling temperature to 28℃” and summer electricity bill.

**Fig. 7:**
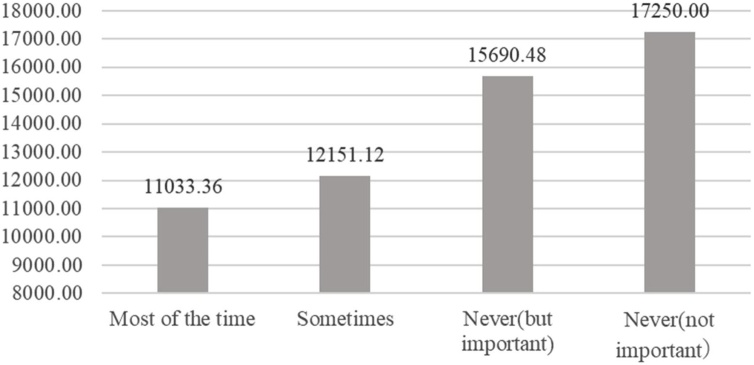
Relation between how often respondents carry out action of “Using curtains and blinds when using air conditioning” and summer electricity bill.

The above correlation can be explored further with reference to the electricity bill for August in ‘an energy-saving use of air conditioning’. This is an important factor to explore further, as the main component of electricity bills in Japan in summer is air conditioning, taking up an average of 58% of electricity bills in 2013 according to Ministry of Environment figures (2020). Fig. 6, Fig. 7 show the average electricity bill according to how often respondents carried out the action of ‘setting the room temperature at 28 °C’ and ‘using curtains and blinds when using air conditioning’.

As for ‘setting the room temperature at 28 °C’’, the average August bill of respondents who carried and did not carry this out on an everyday basis was JPY 10,541 and JPY 13,560, respectively. There was a difference of about JPY 3,000 between the two. As for ‘using curtains and blinds when using air conditioning’, the average August bill for those who carried and did not carry this out was JPY 11,033 and JPY 16,000, or a difference of about JPY 5,000.

As for how air conditioning use and electricity bill varied between households living in apartments and those in detached homes, people livubf in detached houses use air conditioning one hour more than those in apartments during daytime in summer in average. The average electricity bill for detached houses was JPY 14,047 per month, compared to JPY 10,015 for apartments. As the main component of the electricity bill in summer in Japan is the use of air conditioning (Ministry of Environment, 2020), we can say that households living in detached houses almost pay JPY 4,000 more than households living in apartments due to the use air conditioning per month during summer.

### 3.5 Factors influencing mitigation and adaptation behaviour in the use of air conditioning

The previous sections show that there are different approaches to using air conditioning. One is to focus on using air conditioning in a way that reduces energy consumption and thus mitigates climate change; and the other is to use air conditioning primarily to adapt to increasing temperatures. Consequently, to identify factors influencing behaviour, a multiple regression analysis, with ‘an energy-saving use of air conditioning’ and ‘the use of air conditioning’ as criterion variables and perception and situations as explanatory variables, was conducted.

Multiple regression analysis of the statement ‘Reduce use of air conditioner’ (Table 5) showed the action was positively correlated with the explanatory variables ‘difficult to sleep’, ‘due to the obstruction of ventilation’, ‘I think it is a serious situation’, ‘my house has good ventilation’, ‘we should promote “cool share”’, and ‘many opportunities to use public facilities’. ‘An energy-saving use of air conditioning’ was negatively correlated with ‘it is difficult not to use it while others are using it’, ‘I use air conditioning more frequently’, ‘it is difficult to go out during the day’, and gender. The analysis indicate that those who actively use air conditioning in an energy-saving manner are aware of the current environmental problems, actively carrying out environmental behaviours, such as ‘cool share’, and actively using public facilities and going out often.

**Table 5:** Multiple regression analysis of "Reduce use of air conditioner".

Explanatory variable	Coefficient	T value	Significant probability	VIF(Variance Inflation Factor)
Difficult to sleep	.281	3.279	.001	1.922
Due to the obstruction of ventilation	.200	3.015	.003	1.152
I think it is a serious situation	.142	1.975	.050	1.349
My house has good ventilation	.139	2.145	.033	1.106
It is difficult not to use it while others are using it	-.129	−1.911	.058	1.190
We should promote “cool share”	.216	3.279	.001	1.141
I use air conditioning more frequently	-.195	−2.513	.013	1.583
Many opportunities to use public facilities	.180	2.719	.007	1.146
It is difficult to go out during the day	-.193	−2.256	.025	1.919
Gender	-.212	−3.217	.002	1.134

Adjusted R^2^＝0.872 objective variable: Reduce use of air conditioner.

‘The use of air conditioning’ were significantly correlated with ‘it is difficult to use air conditioning appropriately’ and ‘I use air conditioning more frequently’ (Table 6). The variable ‘it is difficult to use air conditioning appropriately’ means that it is difficult to use air conditioning in a way that promotes energy-saving, yet also keeps the home and the people in it suitably cool. In other words, it stands in stark contrast to ‘an energy-saving use of air conditioning’. There is also some confusion about the appropriate use of air conditioning while acknowledging the necessity of energy-saving. This suggests that it is necessary to educate the public about an efficient use of air conditioning and how to use air conditioning while saving energy.

**Table 6:** Multiple regression analysis of " The use of air conditioning.

Explanatory variable	Coefficient	T value	Significant probability	VIF
It is difficult to use air conditioning appropriately	.214	2.895	.004	1.131
I use air conditioning more frequently	.227	3.077	.002	1.131

Adjusted R^2^＝0.138 objective variable: The use of air conditioning.

### 3.6 How distance of homes to green spaces affects use of these spaces and other related items

There was a negative correlation between ‘shade preference’ and the electricity bill for August. Its constituent variables, ‘using a hat and parasol’ and ‘not going out when it is hot’, had a non-significant negative coefficient, whereas ‘staying under the shade of trees’ had a significant negative coefficient. This suggests that when one stays in the shade, one is not at home and, therefore, consumption of electricity for air conditioning and other household appliances is reduced.

Table 7 shows the results of a multiple regression analysis with ‘staying under the shade of trees’ as a criterion variable. Five constants, namely, ‘taking part in a workshop’, ‘there is shade and green spaces in the neighbourhood’, ‘difficult to sleep at night’, ‘difficult to go out during the day’, and ‘I would like to do what others are doing’ were found to be significant. ‘Staying under the shade of trees’ was related to ‘there are green spaces near my home’ and ‘going out during the day’. In other words, it is necessary for the shade of trees to be located near one’s home for it to serve as a place to stay at.

**Table 7:** Multiple regression analysis of "Stay in the shade of trees".

Explanatory variable	Coefficient	T value	Significant probability	VIF
Taking part in a workshop	.329	5.002	.000	1.032
There is shade and green spaces in the neighborhood	.216	3.262	.001	1.047
Difficult to sleep at night	.309	3.678	.000	1.682
Difficult to go out during the day	-.217	−2.638	.009	1.611
I would like to do what others are doing	-.144	−2.112	.036	1.109

Adjusted R^2^＝0.223 objective variable: Stay in the shade of green.

**Fig. 8:**
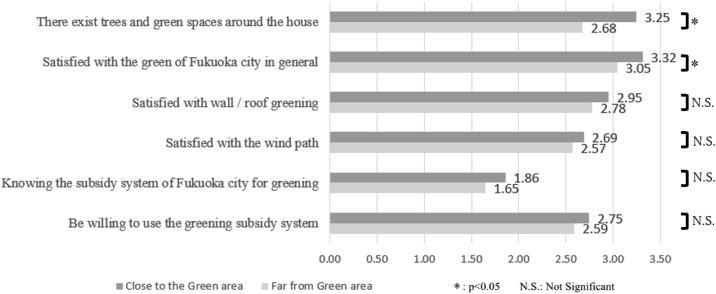
Evaluation of quality of green environment depending on proximity to green space (0=strongly negative evaluation; 4=strongly positive evaluation).

Fig. 8 shows the comparison of mean scores assigned to different aspects of the quality of the urban green environment in Fukuoka, between respondents whose home were near and far from parks/green spaces. ‘There is shade and green spaces near my home’ scored 3.25 among respondents whose homes were close to parks/green spaces and 2.68 among those whose homes were far. ‘I am satisfied with green spaces in Fukuoka City as a whole’ scored 3.31 among respondents whose homes were close to parks/green spaces and 3.05 among those whose homes were far.

**Fig. 9:**
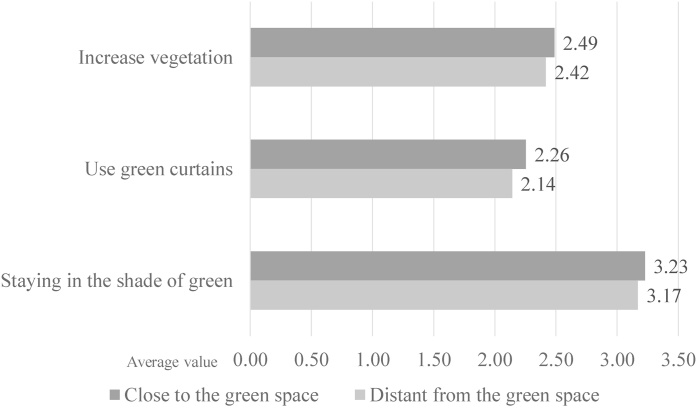
Likelihood of undertaking actions to urban greening depending on proximity to green space (0=strongly negative evaluation; 4=strongly positive evaluation).

Fig. 9 shows the comparison of means of behaviours related to the use of green spaces based on the distance between home and parks/green spaces. ‘To increase vegetation’ (i.e. planting vegetation) scored 2.49 among respondents whose homes were near parks/green spaces and 2.42 among those whose homes were far. ‘To use “green curtains”’ scored 2.26 among respondents whose homes were near parks/green spaces and 2.14 among those whose

homes were far. ‘Staying in the shade of trees’ scored 3.23 among respondents whose homes were near parks/green spaces and 3.17 among those whose homes were far. These differences were not, however, found to be significant in the t-test.

## 4 Discussion

### 4.1 Cooling behaviours and practices

It was notable in our findings that those using air conditioning in an energy saving manner also engaged with a number of other cooling practices outside the home. These included staying in shade, using ‘cool share’ and other public facilities, and going out often. It is notable that some respondents, at least those with green spaces near their homes, still valued the shade of trees as a cooling strategy in spite of the high prevalence of air conditioning in Japan. Building on recent research into practices in more temperate climates where air conditioning and associated cooling behaviours are a relatively new phenomenon, our findings thus remind us of two points. The first is that in the tropics, understandings of cooling practices may already be well established. These may encompass not only a wider basket of individual and household actions extending beyond the use of air conditioning identified in our factor analysis such as shading, fluid intakes and daily rhythms (Chee, Chang, & Wong, 2011; Oppermann, Walker, & Brearley, 2020); but also institutional arrangements such as the provision of shared neighbourhood cooling facilities and community patrols to identify and support the most vulnerable – initiatives which have also taken root in more heat-prone US cities in recent years (Sampson et al., 2013; Sheridan, 2007). The second point is the utilisation of shading and of green spaces as a cooling strategy in Fukuoka, as illustrated in Section 3.6. This relates to existing work in other tropical zone Asian cities (Mabon, 2020; Song, Richards, Edwards, & Tan, 2017) which points to a longer socio-cultural engagement in the tropics with green spaces as a source of shade and cooling, and also the different potential for climate mitigation and adaptation which may be afforded by tropical urban ecosystems. Again, the idea of visiting green spaces as a cooling strategy is gaining interest in more temperate climates (Arnberger et al., 2017; Coccolo, Kämpf, Mauree, & Scartezzini, 2018), but may already be a well-established and familiar practice in locales such as Fukuoka.

As such, our findings reflect the calls of Shove et al. (2014) and Walker et al. (2014) to understand cooling behaviours in relation to their wider cultural and institutional contexts. Our findings show that staying cool is not only a household or domestic practice, but bound up with people’s daily practices and mobilities such as accessing green spaces and public services. Nonetheless, the more limited uptake of efficient and energy-saving uses of air conditioning – and the effect this has on households’ energy bills in summer (Section 3.4.) may call into question the extent to which existing socially and culturally supported cooling practices are able to cope with the pace and extent of climate change. There may thus be a need to supplement existing local knowledges of cooling practices with additional technical input to ensure citizens’ cooling behaviours remain appropriate for the intensity of contemporary climate change and urban heat island effects.

### 4.2 Communication on mitigation and adaptation behaviours

Our findings on the different motivations for and practices of using air conditioning are broadly consistent with existing research. In our results, those who actively use air conditioning in an energy-saving manner (i.e. set to higher temperature, combined with closing blinds) tended to be more aware of environmental issues and engage with a wider range of pro-environmental behaviours. Separately but related, those engaging in energy-saving uses of air conditioning also saw lower electricity bills during the summer period. There are here links to findings from other research (e.g. Howell et al., 2016; Nauges & Wheeler, 2017) indicating thsat whilst promotion of behaviour change in terms of mitigation may be engaging to those already concerned about environmental issues, messaging around a broader suite of adaptation benefits may connect with those with more limited concern or awareness. In the Fukuoka case, the notable difference in energy consumption (and associated financial benefit) may be a pathway to wider engagement on cooling behaviours. A further way to engage households might also be to emphasise that many energy saving/mitigation behaviours are also effective ‘adaptation’methods that allow people to keep cool more effectively, and hence that saving money and using less energy does not come at the cost of keeping cool. This would build on existing ideas of mitigation and adaptation co-benefits at the city level (Puppim de Oliveira, 2013; Thornbush et al., 2013) by extending the idea of practices producing co-benefits to the household scale (Bain et al., 2016).

Nonetheless, our findings also indicate that even in cases where people acknowledge the need for energy saving, there may be confusion over how to use cooling technologies ‘efficiently’ (Section 3.5.) This finding reflects Sampson et al. (2013) and the argument that in addition to simply listing cooling behaviours, some publics may require additional evidence-based guidance as to how to implement these behaviours in practice. In the case of Fukuoka, the message is thus *not* ‘do not use air conditioning’, but rather ‘use air conditioning appropriately’ accompanied by technical and practical guidance on doing so.

Finally, our findings also hint at the benefits of promoting greening practices at the household or neighbourhood level. Our results show that for respondents to be able to benefit from the shade of trees as a cooling strategy, the trees need to be located near to the respondent’s home, and also that satisfaction with the city’s green spaces is higher among those living near green spaces (Section 3.6). On the face of it, neither of these are particularly surprising findings. Yet research elsewhere indicates that participation in neighbourhood-level urban greening practices may bring a range of benefits beyond climate adaptation, including increased social cohesion and higher environmental awareness (Bendt, Barthel, & Colding, 2013; Dennis & James, 2017; Tidball & Krasny, 2011). As such, greater promotion of and engagement on support for neighbourhood and community-level greening within Fukuoka and cities like it may help to facilitate citizen engagement with the wider suite of mitigation and adaptation behaviours discussed in this paper. The *Green Curtain*, *Flower City Fukuoka* and *Flower and Green Urban Placemaking* initiatives led by Fukuoka City Government may be useful starting points for the city in this regard.

### 4.3 Limitations and next steps

Our survey focused on adaptation behaviours in relation to heat and cooling, partly because this is an issue where the interface between mitigation and adaptation behaviours is most apparent due the role of air conditioning, and partly because this is a significant climate risk for Fukuoka. Further research, similar to that of Demski et al. (2017) in the UK context, may wish to consider individuals’ mitigation actions in relation to a wider suite of adaptation behaviours such as awareness of and preparation for floods, typhoons and other extreme weather events. It may be particularly interesting to explore how people in a country such as Japan, with a long history of disaster planning and management, understand adaptation behaviours specific to risks associated with climate change and whether previous experience with disaster prevention and preparation supports adaptation behavours (after Boeckmann, 2016). It is also worth noting that our sample was not representative of the demographics and socio-economic characteristics of Fukuoka as a whole. Whilst our primary aim was to make conceptual contributions to research on citizens’ mitigation and adaptation behaviours rather than to make generalisations about the population of Fukuoka, further research may wish to explore whether behaviours and attitudes differ across gender, age and socio-economic characteristics through wider sampling that is representative of the characteristics of the locality. This could include looking into whether practices differ between respondents in different areas or different types of housing.

Moreover, whilst our study solicited responses across a range of demographics and socio-economic groups, we did not explicitly consider those who may be most vulnerable to extreme heat. With a number of studies in North America and Australasia advocating specific attention to the adaptation actions of the most vulnerable (Byrne et al., 2016; Sampson et al., 2013), further enquiry into the actions necessary to safeguard the most vulnerable in a Japanese context would be a logical extension of this research. However, it should not be assumed that those identified as most vulnerable in a North American, European or Australasian context will also be the most vulnerable in Japan. As a precursor to further research into the engagement of the most vulnerable members of society with adaptation and mitigation actions, further research may wish to consider how local social and cultural practices inform who is vulnerable to climate risks such as heat and how there are cared for.

## 5 Conclusion

In this paper, we assessed citizens’ adaptation and mitigation behaviours – especially cooling practices – in response to the urban heat island effect in Fukuoka, Japan. In doing so, we sought to bring a Japanese subtropical city perspectice to existing research on the societal dimensions of cooling practices, and to offer insights into communication and messaging strategies at the mitigation-adaptation interface. Our findings revealed a contrasting tendency between people focusing on mitigation in their cooling practices (those emphasising energy-saving) and people focusing on adaptation in their cooling practices (those using air conditioning to regulate temperature). Our survey also found there is a lack of knowledge on to how to use air conditioning appropriately in a manner that is also saves energy, and that those engaging in energy-saving behaviours may be more likely to combine air conditioning use with other practices such as staying in shade or visiting cool-sharing facilities. Accordingly, we propose that communication measures to demonstrate more effective uses of air conditioning – for instance, setting appropriate room temperatures and combining with use of shading – may help to reduce the heat island and climate impacts of those focusing on adaptation behaviours. Moreover, communicating the economic benefits of appropriate air conditioning use on electricity bills may help to engage less aware citizens. Finally, however, we note that taking advantage of shade from green spaces may only be possible for those living close to green spaces, hence we suggest the promotion of household or neighbourhood-level greening as a means of enabling a breadth of mitigation and adaptation behaviours.

Conceptually, our findings reinforce and build on emergent thought on the relation between individuals’ cooling practices and the wider social and cultural context, but question the extent to which extant social and cultural practices of cooling may be able to cope with the pace and extent of climate change. In practical terms, our results tend to support what is already known about mitigation behaviours being more engaging to those with high environmental awareness or concern, versus adaptation behaviours and/or wider rationales being more effective with those with lower awareness or concern. We thus argue that a breadth of rationales for actions bringing joint mitigation and adaptation benefits – and the provision of practical information to help citizens modify existing practices – may help to maintain the adaptation benefits of air conditioning in subtropical contexts whilst reducing negative climate change and urban heat island effects.

## Conflict of interest

The authors declare no conflicts of interest associated with this manuscript.

## Declaration of Competing Interest

The authors report no declarations of interest.
